# Custodial and perinatal care patterns of women who received prenatal care while incarcerated in the Arkansas state prison system, 2014–2019

**DOI:** 10.1186/s40352-024-00268-7

**Published:** 2024-04-13

**Authors:** Melissa J. Zielinski, Mollee Steely Smith, Alleigh Stahman

**Affiliations:** Psychiatric Research Institute, University of Arkansas for Medical Sciences, 4301 W. Markham Street, Little Rock, AR 72205 USA

**Keywords:** Incarceration, Prison, Pregnancy, Perinatal care, Drug Addiction, Reincarceration

## Abstract

**Background:**

The extraordinary growth in women’s incarceration over the past several decades has resulted in calls for expansion of research into their unique needs and experiences, including those related to pregnancy and perinatal care. However, while research into the health outcomes of women who are incarcerated while pregnant has grown, research on women’s custodial and perinatal care patterns has remained nearly non-existent. Here, we sought to describe (1) the characteristics of the population of women who came to be incarcerated in a state prison system during pregnancy and (2) the characteristics of women’s custodial and perinatal care patterns during and after incarceration.

**Methods:**

We conducted a retrospective chart review of the population of women who received perinatal care while incarcerated in the Arkansas state prison system over a 5-year period from June 2014 to May 2019. Electronic medical records and state prison records were merged to form our study population. Data were from 212 women (*M*age = 28.4 years; 75.0% non-Latina White) with a singleton pregnancy who received at least one obstetric care visit while incarcerated.

**Results:**

Drug-related convictions were the most common crimes leading to women’s incarceration while pregnant, and violent crime convictions were rare. Nearly half (43.4%) of women who gave birth in custody did so within 90 days of admission and the great majority (80.4%) released within 1-year of giving birth, including 13.3% who released within 30 days.

**Discussion:**

The frequency with which women who became incarcerated while pregnant released from prison either prior to or shortly after giving birth was a striking, novel finding of this study given the implications for perinatal care disruption among a high-risk population and the harms of forced separation from infants within hours of birth.

**Conclusions:**

Diversionary programs for pregnant women convicted of crimes, particularly in states without current access, are urgently needed and should be a priority for future policy work.

## Introduction

Women were the fasting growing incarcerated population in the United States from 1980 to 2019, increasing approximately 550% and at a rate twice as high as men during that interval (Carson, 2022; Zeng, 2022). In 2020, women’s imprisonment fell by 23% due to the COVID-19 pandemic; however, this trend is quickly reversing (Kajstura & Sawyer, 2023). Minority women are disproportionately incarcerated; Black women are incarcerated at a rate 2.3 times greater than White women and Latina women at a rate 1.5 times greater than White women (Grassley et al., 2019). An estimated 61% of women entering prison are mothers (Carson, 2022; Shlafer et al., 2015; Sufrin et al., 2019) and recent estimates suggest 3-6% are currently pregnant—thus, approximately 3,000 women entering prisons and 55,000 women entering jails per year are pregnant (Sufrin et al., 2019; Sufrin, Jones, Sufrin et al., 2020a, b). Women’s incarceration therefore carries with it the need for gender-specific considerations such as how carceral systems will respond to motherhood, pregnancy, and childbirth.

There is a small body of research examining the medical care and health outcomes of women who were incarcerated while pregnant (Baker, 2019; Bell et al., 2004; Carter Ramirez et al., 2020; Knight & Plugge, 2005a, b; Shlafer et al., 2021a; Steely Smith et al., 2024), including how incarceration history (Cordero et al., 1991; Egley et al., 1992; Hessami et al., 2022; Howard et al., 2009, 2011; Kyei-Aboagye et al., 2000) and programming receipt (Bard et al., 2016; Shlafer et al., 2021a; Shlafer et al., 2021b) intersect with birth outcomes. However, research on the custodial and perinatal care patterns of women who become incarcerated while pregnant remains virtually non-existent. The few existing studies that examined the intersection of custody, pregnancy, and perinatal care are dated and focused exclusively on the duration and timing of incarceration during pregnancy in relation to specific outcomes, such as birth-weight (Howard et al., 2009, 2011; Martin et al., 1997a; Martin, Reiger, Martin et al., 1997a, b). For example, Cordero and colleagues (1991) found that pregnant women who were incarcerated for over 120 days were more likely to birth term infants of normal birth weight compared to those with less than 90-day sentences. Martin et al. (1997a, b) found that the greater number of weeks spent incarcerated during pregnancy was positively correlated with increases in infant birth weight. More recent studies examining care quality have raised questions about whether carceral care meets community standards. In a recent systemic review and meta-analysis of prenatal care and pregnancy outcomes among women who were incarcerated while pregnant in the U.S., Hessami and colleagues (2022) found that 34% received inadequate prenatal care when examining the total number of prenatal visits and timing of visits related to weeks of gestation (i.e., fewer than the number of clinically recommended prenatal visits). Together, Hessami et al. (2022) and much of the existing literature focus on the simple number of carceral prenatal visits. Little to no research has examined pregnancy and postpartum acute care utilization during incarceration or time between prison intake, childbirth, and release. Also, to our knowledge, no studies have examined the criminal legal histories of women who are incarcerated while pregnant in-depth, including incarceration before or after the current pregnancy or disciplinary violations during pregnancy and the postpartum period. This information is important to understanding the public health and financial costs versus purported public safety benefits of incarcerating people who are pregnant and/or will give birth in custody.

## The current study

The primary aim of this study was to examine the custodial and perinatal care patterns of women who entered Arkansas’ state prison system while pregnant. Due to the limited knowledge regarding the intersection of women’s pregnancies and the timing of their incarceration, we wanted to understand (1) to what degree women’s incarceration overlapped with their pregnancies and (2) the characteristics of perinatal care provided to incarcerated women. In Arkansas, all people who are incarcerated while pregnant are held within the same state prison facility and all perinatal care—including childbirth services—is provided through a single medical center. This provided a unique opportunity to comprehensively link custodial and perinatal care records during the study period. Our specific, policy-relevant research questions were as follows:

1. What are the criminal legal histories and outcomes of women who are incarcerated while pregnant?
2. How does pregnancy, childbirth, and perinatal care intersect with incarceration, including prison entry and release?

## Method

### Study population

A total of 212 unique women with a singleton pregnancy for which they received at least one obstetric care visit while incarcerated in an Arkansas’ state prison between June 2014 and May 2019 were included in our study population. The mean age was 28.4 years (SD = 5.1 years) and ranged from 18 to 43 years. Most women were non-Latina White (75.0%), consistent with the demographics of the prison during the time that the study occurred; others identified as non-Latina Black (11.8%), Latina (7.1%), or non-Latina and another race (6.1%). The great majority of women had been pregnant at least once before (89.6%); some (10.8%) had a previous pregnancy while incarcerated in the same prison.

Detailed information about the health characteristics and outcomes of the women included in the study population, as well as those of their infants, are reported elsewhere (see Steely Smith et al., 2024). In brief, many women had complex physical health histories and over half had histories of illicit substance use and/or mental illness. Of the 219 singleton pregnancies included in the overall study,^1^ 66.7% resulted in the birth of a live infant while the mother was still in custody, 6.8% resulted in a pregnancy loss in custody (e.g., miscarriage, intrauterine fetal death, ectopic pregnancy), and 26.5% had not concluded prior to release from prison. Here, we report data from each individual’s first incarceration while pregnant, thus the slightly lower sample size.

## Procedure

This study was reviewed and approved by the Institutional Review Board at the University of Arkansas for Medical Sciences. During the study period, all people who were determined to be pregnant upon intake to Arkansas’ prison system were transferred to a single prison where they received their perinatal services from a nearby university hospital system. Data for this study were thus able to be comprehensively aggregated from a combination of prison administrative records and electronic medical records (EMR) from the perinatal service provider. EMRs to be included were identified based on a group of chart indicators including presence of a “prisoner” flag and/or addresses indicative of incarceration. Perinatal care receipt in the clinic associated with the prison was also required.

Data aggregated from the prison administrative records provided by the Arkansas Department of Corrections included: basic demographics; all movements within the prison system (e.g., dates and locations of intakes, transfers, releases); all associated convictions and corresponding administrative data (e.g., date(s), case number(s), county of conviction); and records of all disciplinary violations and associated sanctions incurred. This data was provided for all people who were incarcerated in the state prison to which pregnant people were transferred for at least one day between June 1, 2014, and May 31, 2019, with information current through the date of the data pull.

Data extracted from the perinatal service provider’s EMR included a wide range of information about the current pregnancy; health service receipt; current and prior medical and obstetric history; childbirth information; and infant outcomes (see Steely Smith et al., 2024 for greater detail). For the current study, we largely utilized variables which recorded the dates and counts associated with women’s health service receipt (e.g., perinatal visit dates and counts; admission and discharge dates associated with hospitalization for childbirth).

Information across the prison and EMR sources was matched using available identifiers, which allowed us to (1) calculate new variables of interest (e.g., time from prison intake to prison release, time from prison intake to childbirth) and (2) segment Department of Corrections data with respect to incarceration during pregnancy (e.g., whether particular events such as convictions and disciplinaries occurred prior to, during, or after pregnancy). All analyses were descriptive and were conducted using IBM SPSS Statistics version 28.0.

## Results

The custodial and perinatal care patterns of our study population are summarized in Tables 1 and 2, with descriptives separated into groups based on prenatal care outcome given the impact of these outcomes (i.e., gave birth to a live infant in custody, still pregnant when released from prison, and experienced pregnancy loss in custody) on many of our variables of interest. We also present custodial patterns for the full population.

**Table 1:** Criminal legal histories and outcomes of study population

	Full Population (*N* = 212)	Gave Birth to a Live Infant in Custody (*n* = 143)	Still Pregnant when Released from Prison (*n* = 54)	Experienced Pregnancy Loss in Custody (*n* = 15)
Variable	*n* (%) or *M* ± *SD*	*n* (%) or *M* ± *SD*	*n* (%) or *M* ± *SD*	*n* (%) or *M* ± *SD*
**Conviction(s) leading to current incarceration** ^1^				
Drug	128 (60.4%)	85 (59.4%)	32 (59.3%)	11 (73.3%)
Property	63 (29.7%)	41 (28.7%)	19 (35.2%)	3 (20.0%)
Financial	43 (20.3%)	29 (20.3%)	11 (20.4%)	3 (20.0%)
Revocation	26 (12.3%)	19 (13.3%)	6 (11.1%)	1 (6.7%)
Violent	21 (9.9%)	16 (11.2%)	3 (5.6%)	2 (13.3%)
Obstruction	20 (9.4%)	15 (10.5%)	4 (7.4%)	1 (6.7%)
Child abuse or maltreatment	10 (4.7%)	8 (5.6%)	1 (1.9%)	1 (6.7%)
**Number of previous incarcerations**				
0	95 (44.8%)	67 (46.9%)	23 (42.6%)	5 (33.3%)
1	56 (26.4%)	30 (21.0%)	22 (40.7%)	4 (26.7%)
2	25 (11.8%)	22 (15.4%)	2 (3.7%)	1 (6.7%)
3	19 (9.0%)	14 (9.8%)	3 (5.6%)	2 (13.3%)
4	6 (2.8%)	3 (2.1%)	2 (3.7%)	1 (6.7%)
5+	11 (5.2%)	7 (4.9%)	2 (3.7%)	2 (13.3%)
**Time from prison intake to release from prison** ^2^	277.2 ± 254.3 days	330.2 ± 275.5 days	116.8 ± 47.9 days	373.9 ± 251.8 days
0–60 days	4 (1.9%)	--	4 (7.4%)	--
61–90 days	17 (8.0%)	6 (4.2%)	11 (20.4%)	--
91–180 days	54 (25.5%)	22 (15.4%)	30 (55.6%)	2 (13.3%)
181–365 days	92 (43.4%)	75 (52.4%)	9 (16.7%)	8 (53.3%)
1–2 years	28 (13.2%)	26 (18.2%)	--	2 (13.3%)
2 + years	11 (5.2%)	9 (6.3%)	--	2 (13.3%)
Not yet released by end of study period	5 (2.4%)	4 (2.8%)	--	1 (6.7%)
Unknown (missing)	1 (0.5%)	1 (0.7%)	--	--
**Reincarcerated within 1 year of release**				
Yes	31 (14.6%)	20 (14.0%)	9 (16.7%)	2 (13.3%)
No	176 (83.0%)	119 (83.2%)	45 (83.3%)	12 (80.0%)
Not Applicable (i.e., not yet released by end of study period)	5 (2.4%)	4 (2.8%)	--	1 (6.7%)
**Reincarcerated within study period**				
Yes	70 (33.0%)	50 (35.0%)	15 (27.8%)	5 (33.3%)
No	137 (64.6%)	89 (62.2%)	39 (72.2%)	9 (60.0%)
Not Applicable (i.e., not yet released by end of study period)	5 (2.4%)	4 (2.8%)	--	1 (6.7%)

^1^Because women in our study population could have multiple convictions which led to the current incarceration, conviction categories are not mutually exclusive. Also, one person who gave birth to a live infant in custody was missing conviction data

**Table 2:** Perinatal care patterns of study population

	Gave Birth to a Live Infant in Custody (*n* = 143)	Still Pregnant when Released from Prison (*n* = 54)	Experienced Pregnancy Loss in Custody (*n* = 15)
Variable	*n* (%) or *M* ± *SD*	*n* (%) or *M* ± *SD*	*n* (%) or *M* ± *SD*
**Trimester at First Prison Prenatal Visit**			
First	19 (13.3%)	16 (29.6%)	6 (40.0%)
Second	50 (35.0%)	32 (59.3%)	6 (40.0%)
Third	74 (51.7%)	6 (11.1%)	2 (13.3%)
Unknown (provider unable to estimate)	--	--	1 (6.7%)
**Time from Prison Intake to First Prenatal Visit**	10.00 ± 14.56 days	10.35 ± 6.98 days	11.00 ± 11.99 days
0–7 days	81 (56.6%)	17 (31.5%)	8 (53.3%)
8–14 days	45 (31.5%)	29 (53.7%)	5 (33.3%)
15–30 days	12 (8.4%)	7 (13.0%)	1 (6.7%)
31–60 days	1 (0.7%)	1 (1.9%)	1 (6.7%)
61–90 days	2 (1.4%)	--	--
91–180 days	1 (0.7%)	--	--
Unknown (missing)	1 (0.7%)		
**Time from Prison Intake to Childbirth in Custody**	112.05 ± 65.33 days	--	--
0–7 days	1 (0.7%)	--	--
8–14 days	3 (2.1%)	--	--
15–30 days	10 (7.0%)	--	--
31–60 days	23 (16.1%)	--	--
61–90 days	25 (17.5%)	--	--
91–180 days	53 (37.1%)	--	--
180 days or more	27 (18.9%)	--	--
Unknown (missing)	1 (0.7%)		
**Time Hospitalized for Childbirth in Custody**	2.24 ± 1.41 days	--	--
**Time from Childbirth to Release from Prison**	218.64 ± 276.23 days	--	--
0–7 days	8 (5.6%)	--	--
8–14 days	4 (2.8%)	--	--
15–30 days	7 (4.9%)	--	--
31–60 days	15 (10.5%)	--	--
61–90 days	15 (10.5%)	--	--
91–180 days	41 (28.7%)	--	--
181–365 days	25 (17.5%)	--	--
1–2 years	18 (12.6%)	--	--
2 + years	6 (4.2%)	--	--
Unknown (still incarcerated by end of study period)	4 (2.8%)		
**Number of prenatal visits**	9.22 ± 4.42	6.15 ± 3.07	3.07 ± 2.74
**Number of postpartum visits**	2.45 ± 1.34	--	--

### Custodial patterns

Drug-related offenses were the most common crimes leading to women’s incarceration while pregnant, with 60.4% of the population having a conviction with this classification; this crime type was considerably more common than any other offense (Table 1). Incarceration due to violent crime was relatively rare, as was incarceration due to child abuse or maltreatment, with 9.9% and 4.7% of the population having a conviction with this classification respectively. Notably, nearly half of the population was incarcerated in the state’s prison system for the first time. The great majority of women were serving relatively short sentences, with 78.8% released from prison within one year. Moreover, just 14.6% were reincarcerated in the state’s prison system within the year that followed release, and 33.0% had a record of reincarceration in the state’s prison system considering any time within the study period.

### Disciplinary violations and sanctions

Our examination of disciplinary violations revealed that 75.0% of the study population did not receive any disciplinary violations during their pregnancy. Of those still incarcerated after pregnancy had ended (i.e., following childbirth or loss), 72.6% had no disciplinary violations in the interval between childbirth and release.

The three most common disciplinary violations that were recorded during pregnancy were related to keeping order; 41 women (19.3%) were cited for “failure to obey a staff order,” 29 women (13.7%) for “creating unnecessary noise,” and 17 women (8.0%) for “failure to keep one’s person or quarters within regulation.” Citations related to violence were very rare; there were 4 women (1.9%) who were cited for “provoking or agitating a fight” and 3 women (1.4%) cited for “verbal or written threat or physical assault.” Notably, isolation was used as a sanction in some cases; 4 women (1.8%) were sanctioned to isolation during their pregnancy, with 2 women having been isolated for 15 days and 2 women having been isolated for 30 days.

For those who remained incarcerated following childbirth or loss, the most common violations were the same as those that were recorded during pregnancy (21.5% for “failure to obey a staff order,” 15.2% for “creating unnecessary noise,” and 10.8% for “failure to keep one’s person or quarters within regulation”). There was more variety in citations associated with violence, but overall prevalence remained limited. Three women (1.9%) each were cited for “battery on another resident,” “provoking a fight,” “destruction of property,” and “throwing/ejecting bodily fluids;” 2 women (1.4%) were cited for “verbal or written threat or physical assault”; and 1 woman (0.6%) each were cited for “battery on a staff,” “sexual threats,” and “battery upon another resident without serious injury.” A total of 8 women (5.1%) were recorded as having been sanctioned to time in isolation, including 5 women for 30 days, 1 woman for 45 days, and 2 women for 140 + days.

### Perinatal care patterns, childbirth, and time in prison

Perinatal care patterns generally varied by prenatal care outcome, which would have been determined by a combination of gestational age at prison intake and time remaining in one’s sentence.

#### Women who gave birth to a live infant in custody

Of the 143 women who ultimately gave birth to a live infant in custody, 43.4% delivered within 90 days of prison admission, including 9.8% who delivered within 30 days (*Median* = 106 days; *Range* = 0-261 days). On average, these women received 9 prenatal visits (*Range* = 0–25 visits), with those entering prison in their third trimester receiving an average of 6 prenatal visits (*Range* = 0–11 visits). Notably, 8 women were at or beyond 37 weeks gestation at their first prenatal visit in prison, with the latest being 38.43 weeks gestation. One additional woman had no prenatal visits due to being diverted directly to the hospital for childbirth during her initial transport from the intake prison to the prison where all pregnant women are housed; this individual also released from prison less than 3 months after prison intake. The overwhelming majority of women (88.1%) were seen for their first prison-based prenatal visit within 14 days of prison intake. However, 4 women were seen for their first prenatal visit more than 30 days after intake.^2^

Generally, women who gave birth to a live infant in custody were hospitalized a total of 2 days for childbirth and childbirth recovery. However, 2 women (1.4%) spent less than 24 h hospitalized and 32 (22.4%) spent less than a full 48 h hospitalized prior to discharge. Upon return to the prison, women received an average of 2 postpartum care visits (*Range* = 0–9). Although most women received 2 visits, 19 women (13.3%) received only 1 visit and 6 women (4.2%) received no visits, likely due to being released within a month of giving birth.

Time from childbirth to release from prison ranged from as few as 5 days to 5.4 years, though the great majority of women in this category (80.4%) went on to be released from prison within 1 year of giving birth. Among those who released within 1 year of giving birth, 91 (79.1%) released within 6 months, including 19 (16.5%) who released within 30 days.

#### Women who were still pregnant when released from prison

Women who released from prison while still pregnant were much less likely than those who gave birth in custody to have entered prison in their third trimester of pregnancy; 88.9% were in their first or second trimester. They commensurately had fewer prenatal visits while in custody, having received an average of 6 prenatal care visits (*Range* = 1–15). The great majority (85.2%) were seen for their first prison-based prenatal visit within 14 days of prison intake, and all but 1 woman was seen within 30 days of prison intake.

#### Women who experienced pregnancy loss in custody

Similar to those who did not give birth in custody, women who experienced a loss during incarceration were more likely to enter prison in early pregnancy. Loss most often occurred prior to the 20th week of pregnancy, though two women experienced a pregnancy loss after 20 weeks gestational age, including one after 40 weeks gestation.

### Other care intersections with birthing hospital

While conducting our chart review, data extractors noted that some women’s care at the birthing hospital included services other than those rendered during childbirth; data on these services were also extracted to describe these care intersections more fully. These services included pregnancy-related triage visits and hospital admissions, which were experienced by 22.3% and 4.6% of the overall study population respectively. Triage visits during pregnancy were most common among women who ultimately gave birth to a live infant in custody; 29.4% of women who gave birth to a live infant in custody had at least one triage visit during pregnancy compared to 5.6% of those who released from prison while still pregnant and 13.3% of those who experienced pregnancy loss in custody. Among those who gave birth to a live infant in custody, 10 women (7.0%) had a postpartum triage visit and 7 women (4.9%) had a postpartum hospital admission while still incarcerated.

Chart records also revealed that 29.2% of the study population received emergency care in the birthing hospital either before their incarceration (19.3%) and/or after it (17.0%).^3^ Of the total population, 25.5% had been to the emergency department of the birthing hospital prior to incarceration for non-trauma-related physical health concerns, 6.6% for a traumatic injury or assault, 2.8% for a drug overdose, 2.4% for a suicide attempt, 3.3% for other psychiatric concerns, and 6.1% for another presenting concern.

## Discussion

This study is the most comprehensive report to date on the custodial and perinatal care patterns of a population of women who were incarcerated while pregnant. We found that nearly half of all pregnant women who entered the Arkansas state prison system over the five-year period studied were incarcerated in this system for the first time and that the vast majority went on to be released from prison in less than one year. As is true of women who become incarcerated more generally, the great majority were incarcerated for drug-related crime. Few were incarcerated due to crimes that were designated as violent, and prison disciplinary records revealed that even fewer were recorded as engaging in violent behavior while incarcerated. These top-level findings indicate that many women in this population could have been safely diverted from prison, and a high likelihood that diversion would be more appropriate given the notable lack of evidence-based programs for addiction within Arkansas’ prisons (Horton, 2024).

Relatedly, a particularly striking finding of our study was the frequency with which women who gave birth in custody were released *very* shortly thereafter. Nearly 2 in 3 women with a live birth in custody were released from prison within 6 months of childbirth; one woman released less one week after giving birth. Thus, mothers and their newborn infants incurred the dramatic and often non-reversible implications of birth in custody—including forced separation during a critical time for health-promoting contact (e.g., skin-to-skin, breastfeeding)—due to what amounted to, in most cases, requiring that a new mother serve out very few remaining days of a prison sentence. This requirement comes at the additional cost of disrupting early bonding opportunities and introducing risk for termination of the mother’s parental rights (Gifford et al., 2021); it is also likely to result in infants being raised in caregiving environments marked by psychosocial stress and instability (Pendleton et al., 2022). Diversionary programs or prison nurseries have been used by some U.S. states in an attempt to alleviate or delay the consequences of separation, but the structure and eligibility requirements of these programs are highly variable (Justice-Involved Women & Children, 2023).^4^ Data from the present study support maximally inclusive eligibility criteria for such diversionary programs given the natural occurrence of short sentences and non-violent convictions among those who become incarcerated while pregnant.

Another notable finding of our study was the existence of ultra-brief incarcerations of pregnant women who ultimately released prior to childbirth, including for some women who entered prison during their third trimester. While it may be tempting to consider these women “lucky” to have been released prior to childbirth (i.e., having avoided forced separation from infants), there are yet significant harms possible for women in this group. Specifically, women who were receiving prenatal care prior to incarceration would have experienced at least three interruptions in fetal and maternal monitoring as they moved from the community to jail, jail to prison, and prison back to the community. Women who had not received prenatal care prior to incarceration would have lost prenatal care access upon release, and the likelihood of them being able to establish with a community-based provider, particularly in late pregnancy, is unknown.

Importantly, the negative impact of incarceration on prenatal care was not limited to women who were released prior to childbirth. In our study, nine prenatal visits were the average for women who gave birth in custody; however, many women who entered prison in their third trimester and subsequently gave birth in custody had fewer than the minimum of eight clinically recommended prenatal visits in the third trimester for *non-high risk* or *uncomplicated* pregnancies (American College of Obstetricians and Gynecologists & American Academy of Pediatrics, 2017). Future research on the impact of prenatal care interruptions and quality, more generally, would be useful; however, taken together, the prenatal care patterns highlighted by our findings provide more evidence that diverting pregnant women from incarceration should be the norm rather than the exception. In absence of such diversionary programs, carceral facilities should be required to provide pregnant women who are returning to the community with comprehensive reentry support, including help establishing or reestablishing community prenatal care and other indicated healthcare services (e.g., addiction treatment).

At the systems level, it is important to highlight that the burden of incarcerating pregnant women is already heavy; prisons must cover the costs of childbirth in custody, arrange for infant placements, and provide perinatal care. Future studies aimed at estimating the costs of this care and the downstream effects of infant separation versus community-based diversion and/or treatment would be particularly valuable and policy-relevant additions to the literature. There is also a need for research on the experiences of kinship caregivers (often grandmothers or aunts) who assume custody of infants born to incarcerated mothers and on the outcomes of children born to a mother who is incarcerated. While research on the longer-term outcomes of both mothers and infants would be beneficial, policymakers must not wait for such research to weigh any purported benefit of incarcerating pregnant people and from childbirth in custody against the clear societal harms that result from these practices.

### Limitations

Our study had several limitations, including that our study population was constrained to women in prison within a single state, potentially limiting generalizability. Our study scope was also limited by the available data sources, making us unable to describe the population’s pre- or post-prison perinatal care or broader criminal legal system involvement (e.g., arrest history, length of stay in jail prior to prison intake, incarceration history in other states) more comprehensively. Further, prison intake data was limited by missingness and thus the demographic characteristics we were able to report for our sample were also limited; information on factors that are known to intersect with health outcomes and social needs (e.g., socioeconomic status) would have been valuable if available. Additionally, our study occurred entirely prior to the COVID-19 pandemic, and we are unable to speak to how our results may have differed in the interval since. However, given the dearth of data on our research questions, our findings still have critical policy implications. Finally, while we used a very rigorous search strategy to identify our study population, it is possible that some cases that would have qualified may have still been missed; there was no way to verify our population list as prison administrative data does not include data regarding pregnancy status.

## Conclusions

*Estelle vs. Gamble* (1976) established that all incarcerated persons are entitled to health care for “serious medical needs;” however, adherence to professional standards for perinatal care in carceral settings (e.g., ACOG, 2021; NCCHC, 2020; Sufrin, 2018) is not monitored or enforced, resulting in varied policies, programs, and outcomes across the U.S. (Buchanan, 2012). State and federal initiatives have begun to expand support for women who are incarcerated during pregnancy (Kotlar et al., 2015; Schroeder & Bell, 2005); however services vary widely in their accessibly and provision of care (Shlafer et al., 2022; Wilson et al., 2022). Indeed, it is notable that even amongst the growing body of literature on enhanced perinatal programs in carceral settings (e.g., Wilson et al., 2022), comprehensive programs specifically targeting mental health and addiction amongst this population are rare (Steely Smith, Wilson et al., 2023a; Steely Smith et al., 2023b).^5^

Taken together, our findings and those from past studies at the intersection of incarceration and pregnancy raise critical questions about when and for whom incarceration—rather than diversion to community-based treatment or alternative sentencing—is acceptable. Programs that divert pregnant women who come into contact with the criminal legal system from prisons and jails have tremendous potential to reduce the many harms associated with incarceration and to promote the health of future generations. Until such programs are realized, our work further underscores the need for more and higher quality behavioral health treatment in women’s prisons, as has been voiced for decades (cf. Messina & Esparza, 2022). Without change, the collateral consequences and cyclical harms of relying on incarceration as a response to addiction and its impact on future generations will continue to unfold.

## Data Availability

Portions of the dataset that was generated and analyzed in this study is available from the corresponding author on reasonable request. Variables that could be identifying, including in combination with public records, will be removed prior to sharing.

## Footnotes

1 Some women had multiple pregnancies in custody during the study period.

2 The reason for the lengthy interval between prison intake and first prenatal visit is unknown. In 3 of the 4 cases, women were seen for their first prenatal visit within less than 3 days of arrival at the prison providing prenatal care. Gestational age at first prenatal visit in these cases indicates that women were pregnant prior to prison entry; thus, the delay may have been caused either by delayed transfer to the prison providing prenatal care and/or due to delayed knowledge of the pregnancy.

3 Fifteen women had an emergency care visit in the birthing hospital both before and after giving birth there while incarcerated.

4 For example, in Tennessee, people who give birth in custody may be granted furlough for up to six months for parental bonding—delaying but not preventing separation. Other states (e.g., California, Minnesota, Missouri, New Jersey, Texas, and Wisconsin) fully divert to community-based alternatives though sometimes eligibility criteria are barriers to participation by individuals who would seem to be good candidates for diversion.

5 MOUD continuation during pregnancy if started pre-incarceration is a notable exception, though it is commonly discontinued postpartum (Sufrin et al., 2020).
